# Lower urinary tract symptoms and falls risk among older women receiving home support: a prospective cohort study

**DOI:** 10.1186/1471-2318-13-46

**Published:** 2013-05-15

**Authors:** Kathleen F Hunter, Donald Voaklander, Zoe Y Hsu, Katherine N Moore

**Affiliations:** 1 Faculty of Nursing, University of Alberta, Edmonton Clinic Health Academy, Edmonton T6G 1C9, Canada; 2 Alberta Centre for Injury Control & Research, School of Public Health, University of Alberta, Research Transition Facility, Edmonton, T6G 2E1, Canada

**Keywords:** Falls, Aged, Incontinence, Older women

## Abstract

**Background:**

Although lower urinary tract symptoms have been associated with falls, few studies have been undertaken to understand this relationship in vulnerable community dwelling older adults. The purpose of this study was to describe the relationship over time of falls risk and lower urinary tract symptoms among community based older women receiving home support services.

**Methods:**

A prospective cohort study which took place in an urban setting in western Canada. Participants were 100 older women receiving home care or residing in assisted living with home support services and were followed for six months. Demographic characteristics were collected at baseline, with the Timed Up and Go (TUG), International Consultation on Incontinence Questionnaire Female Lower Urinary Tract Symptoms (ICIQ-FLUTS), and self-report of falls collected at baseline, 3 and 6 months. Descriptive statistics were used to summarize demographic data. Differences between the three visits were analyzed using the Friedman test with post hoc analysis and associations between variables by the Spearman Rank-Order Correlation Coefficient.

**Results:**

One hundred women initially enrolled; 88 and 75 remained at three months and six months. Mean age = 84.3 years; 91% reported at least one urinary symptom at baseline and 35% reported falling in the six months prior to enrollment; 15.9% reported falling between the baseline and three months and 14.6% between three and six months. Mean TUG scores at each time point indicated falls risk (27.21, 29.18 and 27.76 seconds). Significant correlations between TUG and ICIQ-FLUTS (r _=_ 0.33, p < .001; r _=_ 0.39, p < .001) as well as TUG and overactive bladder scores (r _=_ 0.25, p = .005; r _=_ 0.28, p < .008) were found at baseline and three months, but not six months.

**Conclusions:**

The association of lower urinary tract symptoms and falls risk in this group of vulnerable community dwelling older women at baseline and three months has potential clinical relevance. Lack of correlation at six months may be due loss of less robust participants, illuminating the difficulty in following frailer groups over time. Further studies are needed to understand the contribution of urinary symptoms to falls risk, and clinicians should incorporate continence assessment within falls risk assessment.

## Background

Lower urinary tract symptoms (LUTS), such as urgency, frequency, nocturia and incontinence, are a significant but poorly understood risk factor for falls [1-6]. Prevalence of LUTS increases with age, with between 30-50% of older adults experiencing such symptoms [7-10]. LUTS have been associated with decline in physical or cognitive function [11,12], and may represent a marker of frailty among older adults [12].

Urinary urgency, urgency incontinence and nocturia are key LUTS associated with falls in older adults. In one US study of 6,049 community-based women, one or more episodes per week of urgency incontinence were independently associated with fall risk (OR = 1.26; 95% CI 1.14-1.40) [13]. Similar findings were reported for 1016 older home dwelling Finish adults with both urinary urgency (RR = 1.7, 95% CI 1.12-2.45) and urinary incontinence (RR = 1.9, 95% CI 1.29-2.88) [14]. In a prospective study of 405 Taiwanese community seniors, urinary frequency or incontinence were reported more frequently among fallers than non-fallers, and were among six factors that predicted falls risk (OR = 2.13, CI not reported) [15]. In addition to urgency and urgency incontinence, stress urinary incontinence [16] and daytime or nocturnal urinary frequency [17-19] have also been associated with falls risk. Data from a longitudinal health screening program of 1508 ambulatory older men and women revealed that nocturia episodes at least twice nightly significantly increased risk of falls (OR = 1.84; 95% CI 1.05-3.22), and that risk increased dramatically in those reporting more than three episodes (OR = 2.15; 95% CI = 1.04-4.44) [20]. These findings were corroborated by Vaughan and colleagues who reported that three or more episodes of nocturia were associated with an increased fall risk (RR = 1.28, 95% CI = 1.02–1.59) [19].

Many of the community-based studies linking LUTS and falls have included samples drawn from a broad population of older adults that likely included both healthy as well frail and vulnerable older adults. In one study that did focus on frail older women, those with mixed urinary incontinence (stress and urgency incontinence) were 3.05 times more likely to fall (95% CI = 1.01-10.2) than those without [5]. As well, many of the community studies were point prevalence, so that the relationship over time between falls and LUTS is not known. It is suspected that lower urinary tract symptoms in the frail older adult gradually worsen over time but no longitudinal studies have been published that provide data to support this belief [21]. As LUTS are often overlooked in the management of older frail adults, and yet place the person at risk of falls and subsequent serious associated morbidity, it is important to know whether there is a progressive, negative change in LUTS status in this population. Thus the purpose of this study was to describe the relationship over time of falls risk and lower urinary tract symptoms among community based older women receiving home support services.

## Methods

### Design

This was a prospective cohort study with measurements at three time points (baseline, three and six months). The study took place in a large urban centre in Western Canada, and data were collected in the participants’ own home or assisted living apartment. The study was approved by the University of Alberta Health Research Ethics Board and all participants provided written informed consent after a research assistant explained the study to potential participants and checked their understanding of participation using the approach described by Karlawish and Casarett [22].

### Sample

Older women were recruitment through home care case managers and assisted living nurse practitioners/care coordinators. Inclusion criteria were: female, age 70 years and older, able to comprehend English and consent to participate, living within the greater urban area receiving home care services in their own home or in designated assisted living facilities. Exclusion criteria were indwelling or intermittent catheterization, mobility limited to wheelchair or bed-bound; unable to independently consent to participate due to pre-existing assessment of incapacity requiring proxy consent, usually related to dementia, or whom the referring clinicians questioned ability to be able to report symptoms. A sample size of 100 was calculated as sufficient to detect a moderate effect size (power at 0.8, and alpha 0.05). To account for dropouts, a sample size of 120 was to be sought. According to Cohen [23] a moderate effect size for a correlation coefficient is an r = .3. This was calculated for correlation value of lower urinary tract symptoms (measured by the International Consultation on Incontinence Female Lower Urinary Tract Symptoms Questionnaire) and falls risk (measured by the Timed Up and Go test).

### Measures

#### International consultation on incontinence female lower urinary tract symptoms questionnaire (ICIQ-FLUTS)

ICIQ-FLUTS is a 12 item questionnaire derived from the Bristol Female Lower Urinary tract questionnaire that measures female lower urinary tract symptoms and symptom bothersomeness (urinary-related quality of life) [24], with 0 being no symptoms and 48 being severe overall filling/voiding/incontinence symptoms. Bother of each symptom is measured on a Likert scale of 0 (not at all) to 10 (a great deal). In initial psychometric analysis, construct validity was found to be good and criterion validity acceptable (κ = 0.29-0.79 for frequency/volume data). The reliability was good with high internal consistency (Cronbach’s α = 0.78) and stability was excellent (r = 0.86 and 0.90) [24]. We used the four questions on the ICIQ-FLUTS that correspond with the overactive bladder symptoms of urgency, urge incontinence, daytime frequency and nocturia to derive a rating of overactive bladder (OAB) symptoms and bothersomeness.

#### Timed up and go (TUG)

TUG is a test of functional mobility which involves timing the person as they rise from an arm chair, walk three meters, turn, walk back and sit down. Scores on the TUG correlate well with the Berg Balance Scale (*r* = -0.72) and the Barthel Index, a measure of activities of daily living (ADL) (r = -.51) [25]. Inter-rater reliability has been judged as good, with an intraclass correlation coefficient of 0.99 in two studies (n = 99 and 60 respectively) [25,26]. Normative mean value for performance of the three meter TUG in adults 70–79 is 8.54 seconds [27]. In the original testing, those who took more than 30 seconds to complete the TUG were more dependent in transfers and ADLs and were unable to venture outside on their own [25]. Where the TUG takes greater than 14 seconds to complete, both sensitivity and specificity in predicting falls were 87% [28].

### Data collection

Data was collected at three time points: at baseline, three, and six months post enrollment. Short time periods were selected because the population was potentially frail and longer time frames might be affected by changes in circumstances (i.e. admission to long-term care, death). At the baseline home visit after consent was obtained, demographic data was collected, ICIQ-FLUTS questionnaire and TUG were completed. Participants who normally used assistive devices (walker, cane) used these for completing the TUG. At each visit, participants were screened for clinical symptoms consistent with a possible urinary tract infection (UTI) in an older person (dysuria, flank pain, suprapubic pain, fever, cloudy urine, urine with foul odor, recent alteration in mental status or functional decline, new onset incontinence) [29] which could have influenced reporting of LUTS. Women who reported symptoms of UTI or requested continence assessment were referred to their family physician or case manager. We also asked about any hospitalizations and use of an indwelling catheter during hospitalization.

At three and six months ICIQ-FLUTS and TUG were repeated as well as an inquiry on the number of falls participants had experienced since the previous visit and a description of any potential contributors to worsening LUTS.

### Data analysis

Data was analyzed using PASW 18.0 (IBM, Armonk, New York). Descriptive statistics were used to summarize demographic data, medical history, questionnaire scores and time to complete the TUG. As the LUTS, derived OAB and TUG data were not normally distributed, we elected to use nonparametric statistical analysis. Differences between the baseline, three month and six month visits for LUTS, the derived OAB score and the TUG scores were analyzed using the Friedman test, a nonparametric alternative to the repeated measures ANOVA, with a p-value of 0.05 or less considered significant. For any statistically significant results, post hoc analysis was conducted by first adjusting the critical alpha value using the Bonferroni correction, with the critical alpha set at .017 (α/κ with κ = the number of time periods .05/3 = .017). Next, we conducted the Wilcoxon test for each possible combination of pairs (baseline-three months, baseline –six months and three months-six months) [30,31]. We used the non-parametric Spearman Rank-Order Correlation Coefficient to examine associations between variables (p ≤ 0.05), and interpreted the correlations coefficients using Cohen’s conventions [23] (.10 small, .30 moderate, .50 large). Differences between independent samples (those remaining in the study at six months, those who had withdrawn at six months) were examined with the non-parametric Mann Whitney *U* test.

## Results

**Figure 1:**
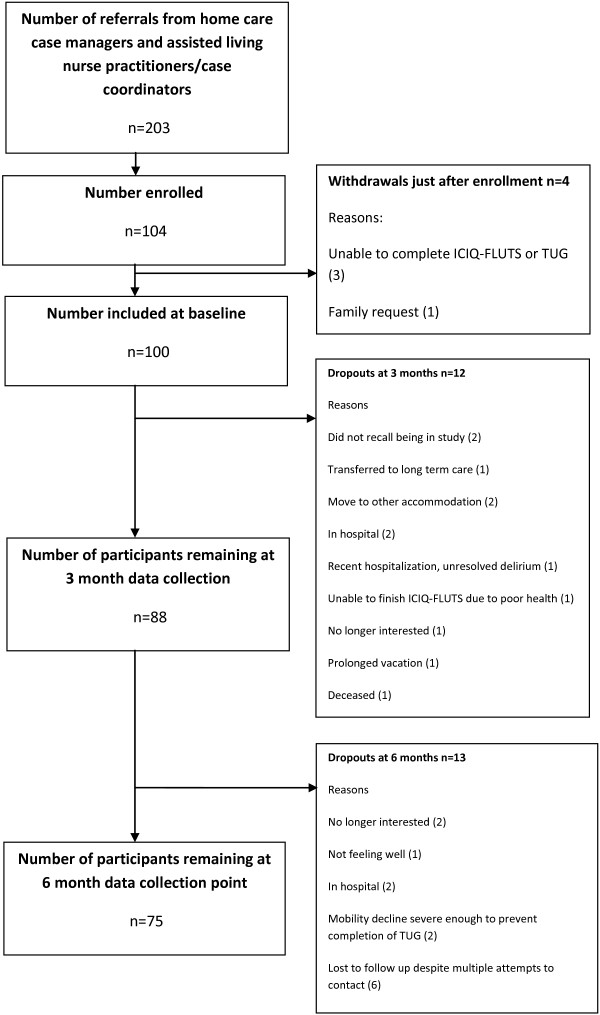
Flow chart of referrals, enrollment and dropouts.

Between October 2009 and June 2011 203 women were referred to the study, with most referrals coming from assisted living. After this time recruitment was stopped as no further referrals were obtained from the home care case managers and assisted living nurse practitioners/care coordinators, and the funding period of the study was drawing to a close. Of these, 104 consented to participate although 4 withdrew just after enrolling (unable to complete the ICIQ-FLUTS questionnaire or the TUG (3), family request (1)), leaving a final sample of 100. At three months, 88 women remained in the study with various reasons for drop outs: did not recall being in study (2), transferred to long term care (1), moved to other accommodation (2), in hospital (2), recent hospitalization with an unresolved delirium (1), unable to finish the ICIQ-FLUTS questionnaire due to poor health (1), no longer interested (1), prolonged vacation (1), deceased (1). At six months, an additional 13 had dropped out leaving 75 participants at the six month time point. Reasons for withdrawal at the final time point were: no longer interested (2), not feeling well (1), in hospital (2), mobility decline severe enough to prevent completion of the TUG (2), lost to follow-up despite multiple attempts to contact (6). Referrals, enrollment and dropouts are summarized in Figure 1. To check for differences between those that withdrew and those that remained in the study at six months, we compared the TUG scores of these groups. Mean TUG score at baseline for those remaining in the study at six months was 24.71 (median 23; SD 11.29), while the mean TUG score at baseline for those who withdrew by six months was 34.72 (median 29; SD 18.82). There were significant differences between these two groups for baseline TUG medians (p = .038) and distribution (p = .006).

Mean age of participants was 84.3 years (SD 7.09), with 35 of the 100 participants self-reporting falls in the six months prior to enrolment. All received some type of supportive in-home care, with 86 receiving services at least daily. The most common services were medication prompting/administration 73 participants), assistance with bathing (56 participants) and assistance with dressing (23 participants). Of medical conditions known to affect bladder function, the most frequently reported was a previous stroke (43), followed by diabetes mellitus (16), and pelvic organ prolapse (12). One participant reported Parkinson’s disease, 43 had undergone hysterectomy, 13 anti-incontinence surgery and 3 had been fitted for a pessary which was still worn. Constipation was rare; 86 of the 100 participants reported daily or at least three times weekly bowel movements. All participants could mobilize independently with or without a walking aid. At baseline, 53 participants used mobility aids, with 49 using walkers and 4 using canes. None of the women reported needing toileting assistance.

Eleven (12.5%) reported having been hospitalized between baseline and three months and another 7 (9.3%) between three and six month visits. Two had an indwelling urinary catheter in hospital, and one reported having a urinary tract infection during hospitalization.

### Lower urinary tract symptoms (ICIQ-FLUTS)

At the initial interview, 91% reported at least one lower urinary tract symptom: leakage of urine before getting to the toilet (urgency incontinence) (66%), needing to rush to the toilet (urinary urgency) (46%), leaking urine when physically active, on exertion or with coughing or sneezing (stress incontinence) (45%), or nocturia more than once a night (39%). New onset of LUTS was reported by three (nocturia or voiding symptoms) at three months and by another four (nocturia, rushing/leaking, voiding symptoms) at six months leaving only two without symptoms for the entire six months in the study. No participants reported resolution of symptoms. Although mean scores for symptom severity tended towards the low end of the scale, there was wide variability in range. Mean scores on the ICIQ-FLUTS and OAB scores for symptom severity and bothersomeness are provided in Table 1. There were no statistically significant differences between overall ICIQ-FLUTS or derived OAB symptom severity scores between the time periods for the n = 75 participants who were in the study at all three time points.

**Table 1:** ICIQ-FLUTS and OAB symptom severity, bothersomeness, nocturia and incontinence (urgency, stress) scores

	**Baseline N = 100**			**Three months N = 88**			**Six months N = 75**		
	**Mean**	**SD**	**Range**	**Mean**	**SD**	**Range**	**Mean**	**SD**	**Range**
ICIQ-FLUTS symptom severity (0–48)*	9	6.93	0-28	7.9	6.49	0-30	7.96	6.22	0-26
ICIQ-FLUTS bothersomeness (0–120)**	9.67	16.54	0-70	8.07	11.82	0-55	9.88	16.40	0-63
OAB symptom severity (0–16)***	4.06	2.95	0-16	3.9	2.92	0-11	4.17	3.21	0-12
OAB bothersomeness (0–40)****	4.6	7.70	0-30	4.24	6.12	0-22	5.35	8.22	0-33
Nocturia episodes nightly*****	1.51	1.25	0-4	1.52	1.22	0-4	1.52	1.20	0-4
Urgency incontinence ******	1.25	1.20	0-4	1.13	1.363	0-4	1.25	1.33	0-4
Stress incontinence ******	0.81	1.09	0-4	0.90	1.23	0-4	0.79	1.06	0-4

*0 = no symptoms, 48 = severe overall filling/voiding/incontinence symptoms; ** 0 = not at all bothered, 120 bothered a great deal; *** 0 = no OAB symptoms, 16 = severe OAB symptoms; **** 0 = not at all bothered, 40 = bothered a great deal; Nocturia episodes: 0 = none, 1 = one, 2 = two, 3 = three, 4 = 4 or more times nightly; Urgency and Stress incontinence: ***** 0 = never, 1 = occasionally, 2 = sometimes, 3 = most of the time, 4 = all the time.

### Falls and risk of falls

At baseline, thirty-five (35%) of participants reported that they had experienced a fall in the six months prior to study enrollment, range from 1 to 8, mean of 1.91 (SD 1.44). Two participants reported fractures (one hip fracture, one pelvic fracture) as a result of these falls. The most common reason given for falling was a slip or trip. Only two connected their falls to toileting needs, reporting that they had fallen when getting up at night to urinate. Table 2 lists reported falls in the six months prior to study enrollment. Of the 88 women remaining in the study at three months, 14 (15.9%) reported falling between the baseline and three months. No injuries were reported, but one participant was seen in the emergency department for blood pressure management after blacking out. Seven women reported one fall, six women reported two falls and one participant fell three times. Eight of these women also reported falls at baseline. Between the three month and six month visits, 11 of the remaining 75 participants (14.6%) reported falling with eight reporting a single fall and three reporting two falls. No injuries or hospital visits were reported resulting from these falls. Eight had reported falls at either the baseline or the second visit, and two reported recurrent falls at all three data collection points.

**Table 2:** Self reported falls

	**Baseline: falls in the six months prior to enrollment n = 100**	**Falls between baseline and three months n = 88**	**Falls between three month and six month follow-ups n = 75**
**Number of participants reporting falls**	35 (35%)	14 (15.9%)	11 (14.6%)
**Number of falls reported per participant who fell**			
**Range**	1-8	1-3	1-2
**Mean**	0.67	0.26	0.63
**SD**	1.25	0.19	0.48
**Reasons participants gave for falls**			
Slip/trip	11	3	7
Dizzy/weak	2	0	1
Legs gave out	3	0	0
Blacked out	1	1	0
Getting up at night to get to toilet	2	2	0
Lost balance	6	2	0
Couldn’t remember	6	3	1
Other	4	3	2

Falls risk, as assessed by the TUG test, was high with mean baseline, three and six month scores at 27.21 seconds, 29.18 seconds, and 27.76 seconds respectively (Table 3). Ninety-two percent of women had a TUG test score of 14 seconds or great at baseline, indicating risk for falls, with only 8% of the women scoring less than 14 seconds (not at risk for falls). At the three months and six months respectively, 89.8% and 90.8% had a TUG score of 14 seconds or greater.

**Table 3:** Mean timed up and go (TUG) scores

	**Baseline N = 100**			**Three months N = 88**			**Six months N = 75**		
	**Mean**	**SD**	**Range**	**Mean**	**SD**	**Range**	**Mean**	**SD**	**Range**
**TUG (in seconds)**	27.21	14.15	10-93	29.18	17.58	10-114	27.76	13.51	9-76

TUG ; >14 seconds = falls risk.

Differences between TUG scores were tested with the Friedman test for the n = 75 participants who were in the study at all three time points. For this group, TUG scores were 24.71 seconds (SD = 11.296), 28.67 seconds (SD 18.444) and 27.76 seconds (SD 13.508) respectively, showing an increase with wider variability at three months and a slight decline with less variability at six months. There was a significant difference in TUG scores between visits (*χ*^2^ = 6.315, df = 2, p = .043). Post hoc analysis of each comparison using the critical alpha of .017 (adjusted by Bonferroni correction) revealed significant differences between the TUG at baseline and three months (p = .005) as well as baseline and six months (p = .004), but no difference in the TUG score between three and six months (p = .373).

Rank correlations between the TUG scores, urinary symptom severity scores (ICIQ-FLUTS and OAB) and number of reported falls are presented in Table 4. At baseline and three months there were statistically significant positive moderate [23] correlations between urinary symptom severity and falls risk, as measured by the TUG. The six month correlation was not significant. There were significant correlations between TUG score and number of reported falls at all three time points.

**Table 4:** Rank correlations TUG, urinary symptom severity and number of reported falls

	**Visit 1**		**Visit 2**		**Visit 3**	
	**N = 100**		**N = 88**		**N = 75**	
**Variables tested**	**r**	**p**	**r**	**p**	**r**	**p**
**TUG and ICIQ-FLUTS total score**	0.33	< .001*	0.39	<.001*	0.21	.075
**TUG and OAB score**	0.25	.005*	0.28	.008*	0.21	.075
**TUG and number of reported falls**	0.22	.007*	0.29	.003*	0.23	.023*

TUG Timed up and go test. * Statistically significant p = .05, Spearman Rank-Order Correlation Coefficient.

## Discussion

Lower urinary tract symptoms are an established falls risk for the older adult. In this study we assessed, over time, the presence of LUTS and number of falls in older community dwelling women. LUTs were reported by 91% of participants; we demonstrated a significant association between overall LUTS and the symptoms that comprise falls risk at baseline and second (three month) follow up visit. There was no significant association of LUTS and the TUG at the six month visit, which may be due to the 25% attrition rate by the six month visit. Although the association between falls and urinary symptoms has been identified in other studies of community dwelling older adults [3-5,13,14,16,32] and in a systematic review [33], only two [5,14] have focused on more frail or dependent community populations. Our study contributes additional data on the association of LUTS and falls risk among older women who have some degree of dependency, rather than a general population of older women where there would be more heterogeneity in terms of independence in activities of daily living.

The participants were at high risk for falls, as seen in the high mean TUG scores at all three data collection points. Only 8% of participants scored less than 14 seconds (low or no risk of falls) at baseline. Thirty-five (35%) of participants reported having had at least one fall in the six months prior to enrolling in the study, and over 14% reported a fall between baseline and the second visit as well as the second and third visit. Although falls among our participants were common, but it is difficult to compare to other published studies. Population based reports of falls rates among community dwelling older people in developed countries vary from 30-60% per year [3,34-36]. Few studies of home care recipients have been undertaken, although Cesari and colleagues [36] reported a 35.9% prevalence of falls within 90 days of admission to a home care program. Reported falls rates vary due to differences in study methods, including sample characteristics, definitions of falls and reporting period, although fall rates are observed in increase with age [37,38].

It is of particular interest that although nocturia, urgency and urinary incontinence (stress or urgency) were commonly reported symptoms in almost half of participants, few reported falls as having been connected to the need to get to the toilet or the presence of any of these urinary symptoms. Several potential explanations exist for this. First, one must of course acknowledge the possibly that the *association* of falls and urinary symptoms in our study and other reports is completely spurious, merely the concomitant presence of two common geriatric syndromes with no cause and effect relationship between them. An alternative explanation is that the relationship is neither spurious nor causal, but arises from some other shared predisposing factors for geriatric syndromes such as lower extremity or sensory impairment [39] or frailty [40]. Divided attention has been suggested as a possible causal explanation between urinary symptoms and falls [33], although this proposition requires further theoretical explanation and testing. The design of the study, a prospective cohort study with measurements at three time points, did allow us to test for associations between LUTS and falls risk, but not for a causal relationships between these variables. To determine the contribution of LUTS to falls, prospective studies with larger samples that include multiple risk factors and include regression analysis, need to be conducted. Including falls and falls risk as endpoints in randomized controlled trials of interventions for LUTS in older women would help to determine if a causal relationship exists.

Self-reported prevalence of LUTS and bothersomeness of those symptoms varied in individual participants. At baseline, three months and six months, mean ICIQ FLUTS scores were low and at each of these time points a small number of participants reported no symptoms. At the opposite extreme a few reported moderate to severe symptoms that again varied in terms of bothersomeness. This variation is not unexpected given that LUTS, and particularly incontinence, are not a normal part of ageing but rather a geriatric syndrome influenced by the presence of pathology, structural changes and functional ability.

This study is limited as the sampling approach involved a voluntary convenience sample. Participants may not represent the population of all older women receiving home support services. As we were not approved to collect data on those who declined, it is not possible to determine if there was a selection bias in our sample in terms of including those that were more or less healthy than those who declined participation. We experienced a high dropout rate, likely related to the age and potential frailty of many of our participants, which limited our analysis and contributed to the study being underpowered. Lack of significant association between falls risk and LUTS at the six month visit may be due to survival of more robust participants. Analysis comparing baseline TUG scores of those who remained in the study at six months and those who had dropped out showed significant differences in median scores and distribution of scores. There was an increase in the TUG mean score, with a wider range and higher standard deviation at the second visit, but some of the women with higher scores dropped out by visit three. As well, the frequency of reporting of most lower urinary symptoms declined by the third visit which may also be the result of frailer women with more symptoms leaving the study. Research with larger samples of older women with dependency in activities of daily living and functional limitations is needed to validate our findings.

A second limitation is that we did not collect data on whether participants had received specialized falls or continence assessment prior to or during the study. We had planned to collect data on medications which would have provided additional insight into falls risk or whether participants were being treated for LUTS, but changes to medication management systems and processes in the assisted living centres severely limited our access to this information as we did not have access to medical records. Similarly, we were not able to obtain data on reasons for hospitalization, length of stay or validate the reports of catheterization or UTI treatment during hospitalization.

Finally, we attempted to exclude those with recognized cognitive impairment, but some of our participants had difficulty recalling details of the fall circumstances weeks or months after the event. Although little difference has been demonstrated between retrospective interviews on number of falls compared to prospective falls diaries [41], a falls diary in which the participants recorded whether their falls occurred in conjunction with toileting or LUTS, rather than relying on recall, might have shed further light on the fall circumstances.

Our recommendations for research are that future studies need to include measurement of cognitive function. Although none of our participants had a known diagnosis of dementia, it may have been valuable to screen for mild cognitive impairment, as it is a potential contributor to falls risk [42] and may have played a role in recall of the contextual details or the reported falls. Including a measure of ADL function would have provided more detail on the physical function of participants. As well a measure of falls risk that incorporates divided attention, such as a TUG that incorporates a duel task, may be more a more sensitive tool to incorporate as rushing to the bathroom might involve divided attention [1] and the cognitive challenge of performing multiple tasks at once [33], such as walking while trying to control the bladder. Further research will be needed to understand the role these variables play in this population. For clinicians in practice, our results show that LUTS are common in older women receiving home support and that there is an association of LUTS and falls risk. Thus we recommend in keeping with current guidelines [43,44], a continence assessment should be a part of the overall assessment of falls risk in frail older women.

## Conclusion

In conclusion, there are moderate, but potentially clinically relevant correlations of falls risk with LUTS and OAB in this sample of vulnerable older women receiving home support services. Future studies should focus on frail women with known LUTS and falls risk in order to further examine this relationship. Such studies need to include measures of other falls risk factors to determine the proportion of variance accounted for by urinary symptoms, as well as explore the possibility that phenomena such as divided attention may play a role in explaining the association of LUTS and falls. Clinicians should incorporate continence assessment within falls risk assessment.

## Abbreviations

LUTS: Lower urinary tract symptoms; OAB: Overactive bladder symptoms; TUG: Timed Up and Go; ICIQ-FLUTS: International Consultation on Incontinence Questionnaire Female Lower Urinary Tract Symptoms; ANOVA: Analysis of variance.

## Competing interests

KFH – Co-investigator on a study funded by Astelles (unrestricted grant) and a study funded by Pfizer (unrestricted grant).

## Authors’ contributions

KFH initially conceptualized the study and collaborated with KNM and DV on design. KFH and ZYH recruited and collected data and conducted the analysis. All authors participated in interpretation of data and preparation of the manuscript. All authors read and approved the final manuscript.

## Pre-publication history

The pre-publication history for this paper can be accessed here:

http://www.biomedcentral.com/1471-2318/13/46/prepub
